# Novel secretion modification region (SMR) peptide exhibits anti-metastatic properties in human breast cancer cells

**DOI:** 10.1038/s41598-022-17534-z

**Published:** 2022-08-01

**Authors:** Ming-Bo Huang, Dara Brena, Jennifer Y. Wu, William W. Roth, Samuel Owusu, Vincent C. Bond

**Affiliations:** 1 Department of Microbiology, Biochemistry, and Immunology, Morehouse School of Medicine, Atlanta, GA 30310 USA; 2 Columbia College, Columbia University, New York, NY 10027 USA

**Keywords:** Cancer, Drug discovery, Molecular biology

## Abstract

Breast cancer is the second leading cause of cancer-related mortality in women worldwide, with nearly 90% attributed to metastatic progression. Exosomes containing epithelial–mesenchymal transition (EMT) ‘programs’ transmit pro-metastatic phenotypes. Our group discovered and developed a novel anti-cancer SMR peptide that antagonizes breast cancer cell exosome release resulting in cell cycle arrest and tumor growth suppression. This study aims to evaluate the anti-metastatic capabilities of the SMR peptide, focusing on exosomes and EMT. Breast cancer cell lines MDA-MB-231 and MCF-7 were treated with the SMRwt peptide, and the following assays were performed: cell wound-healing, migration, invasion. The SMRwt peptide consists of the following amino acid sequence VGFPVAAVGFPVDYKDDDDK and contains the SMR domain (^66^VGFPV^70^) of the HIV-1 Nef protein. Western blot analysis detected epithelial and mesenchymal markers to evaluate EMT progression. Extracellular vesicle type and quantity were assessed through NanoSight analysis. Mortalin and Vimentin knockdown was achieved through antibody targeting and miRNAs. Data gathered demonstrated that the SMR peptide interacts with Mortalin and Vimentin to inhibit pro-EMT exosome release and induce EMT tumor suppressor protein expression. Specifically, SMRwt treatment reduced mesenchymal markers Mortalin and Vimentin expression, while the epithelial marker E-cadherin expression was increased in breast cancer cells and breast cancer-derived exosomes. The SMR peptide specificity was identified as no effect was observed for MCF-10A exosome release or function. Direct Mortalin knockdown paralleled the results of SMR peptide treatment with an effective blockade of breast cancer cell migration. Conversely, the invasion assay differed between breast cancer cell lines with invasion blocked for in MCF-7 but not in MDA-MB-231. These results reinforce the therapeutic value of targeting breast cancer exosome release and reinforce Mortalin and Vimentin as critical regulators and therapeutic targets in breast cancer cell progression, EMT, and metastatic potential. A greater understanding of the SMR peptide mechanism of action will benefit the therapeutic design of anti-metastatic agents.

## Introduction

Breast cancer metastatic progression is a multi-step process that includes local cell invasion, intravasation, dissemination via circulatory and/or lymphatic systems, extravasation, and colonization of distant sites^1^. These sequential steps of breast cancer metastasis are directed, specific, and predictable^2^. Breast cancer cells likely have innate abilities towards malignancy; however, the degree and timing of invasion and metastasis may vary due to the tumor genetic and epigenetic heterogeneity and extrinsic signaling pathways related to the epithelial–mesenchymal transition (EMT)^3^. EMT is a transdifferentiation process whereby stationary epithelial cells adhered to the solid tumor shift towards a motile fibroblastic mesenchymal phenotype^4^. The role of EMT in breast cancer has been demonstrated via numerous in vitro studies in normal and malignant mammary epithelial cells and via in vivo studies using mouse models of breast cancers^5,6^. Increasing evidence indicates an inductive tumor microenvironment at the tumor edge in the facilitation of aberrant EMT activation that leads to breast cancer invasion and migration^7^. Exosomes transmit pro-EMT programs and are highly involved with pro-metastatic intercellular coordination of breast cancer cells with the surrounding tumor microenvironment^8^.

### Role of tumor derived extracellular vesicles (EVs) in promoting migration and invasion through the transmission of pro-EMT programs, chemokines, and degradative enzymes

**Figure 1:**
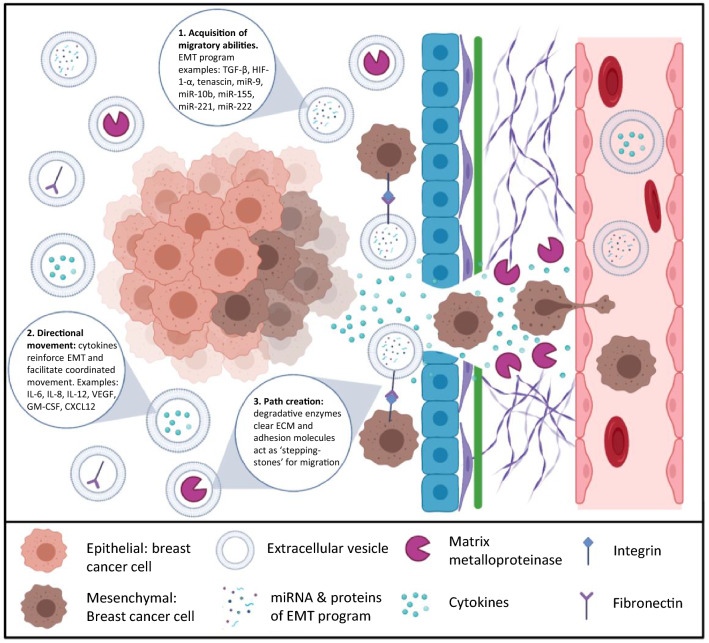
Tumor derived extracellular vesicles (EVs) promote breast cancer cell migration and invasion. (1) Acquisition of migratory abilities. The cargo of tumor derived EVs is frequently referred to as a pro-EMT program whose transmission throughout the tumor population drives an aggressive phenotype. (2) Directional movement. EV release of chemokines encourages movement of breast cancer cells along the path most conducive with breast cancer spread. (3) Path Creation. Degradative enzymes within EVs rapidly remove ECM to clear a path for breast cancer migration and invasion. EVs have been paralleled to ‘stepping-stones’ as they adhere via heparan sulfate and express fibronectin that can be bound by integrins on the surface of migrating breast cancer cells. Created with BioRender.

Extracellular vesicles (EV) are secreted membrane vesicles (30–100 nm in diameter) that act locally and systemically to carry parental cancer cells’ many different molecules such as mRNA, miRNA, DNA, lipids, and proteins^9,10^. EV are critical to many breast cancer mediated processes such as angiogenesis, immune suppression and evasion, extracellular matrix degradation, and metastasis^11–17^. The three primary mechanisms that underly EV elicited breast cancer migration and invasion are (1) transmission of pro-EMT migratory phenotypes throughout tumor populace, (2) coordinated movement through chemokine release, and (3) degradative enzyme pathway creation for migratory cancer cells (Fig. 1). The pro-EMT ‘program’ of tumor derived EV includes, but is not limited to the following: TGF-β, HIF-1-α, tenascin, miR-9, miR-10b, miR-155, miR-221, miR-222^18,19^. TGF- β and HIF-1-α are inducers of EMT that act through the SMAD pathway to increase N-cadherin, decrease E-cadherin, and upregulation of transcription factors TWIST1 and ZEB1/2^18,20^. MiR-9 and miR-10b are associated with heightened Vimentin and absent E-cadherin expressions^21^. miR-155, miR-221, and miR-222 have been linked to EMT driven chemoresistance^22,23^. For directional movement, Migrasomes are a newly defined category of EV secreted along the leading edge of a tumor and contain chemokines strongly related to migration, such as CXCL12^24^. Other cytokines identified by Dalla et al. concentrated in breast cancer EV are IL-6, IL-8, IL-12, VEGF, FGF basic, G-CSF, and GM-CSF^25^. EV transport cytokines and can trigger proinflammatory cytokine production in recipient cells^26^. Before acquiring motility and coordination in movement, tumor derived EV act to transport degradative enzymes resulting in a loss of cell adhesion and ECM digestion to generate a pathway for migration^24,25^. In addition, Chin et al. proposed a mechanism whereby EV act as ‘stepping-stones’ for migrating breast cancer cells through integrin-fibronectin interactions^27^. The importance of EV transport in breast cancer progression, migration, and invasion renders EV a potential novel class of therapeutic targets for inhibition^17,28–30^. Blockade of tumor derived EV by SMRwt peptides may hold promise in identifying other metastatic contributory therapeutic targets and aid anti-metastatic drug development.

### Therapeutic potential of SMR peptide mediated EV inhibition

Exosome-based communication networks represent a promising therapeutic target for inhibition. Our lab developed an anticancer peptide derived from the Secretion Modification Region (SMR; ^66^VGFPV^70^) of the HIV-1 Nef protein. The Nef SMR sequence interacts with cellular proteins, including mortalin and vimentin, and blocks breast cancer cell exosome release. Mortalin belongs to the heat shock protein (HSP) 70 family and acts as a highly conserved molecular chaperone encoded by the nuclear gene HSPA9B. Many studies have reported Mortalin as strongly related to the induction and/or upregulation of EMT, carcinogenesis, and metastasis^31–36^. Mortalin is known to trigger pro-metastatic tumor derived exosome release. However, the specific mechanism of Mortalin’s role in regulating endosomal trafficking remains unclear^37–40^. Vimentin is a 57 KD mesenchymal marker type III intermediate filament that maintains cell integrity and is involved in cell migration, motility, and adhesion. Overexpression of Vimentin in solid cancers drives EMT, invasion, and metastasis^4^. In vitro studies have demonstrated that the knockdown of Vimentin impairs cell attachment, migration, and invasion in breast and colon cancer cell lines^41^. In our previous research, SMR peptide targeting antagonized breast cancer cell proliferation, arrested the cell cycle, and restored complement mediated cell death^42^. To better understand the therapeutic potential of the SMR peptide, we aim to explore the mechanism of SMR mediated inhibition on breast cancer EMT, invasion, and migration.

## Materials and methods

### Cell cultures and antibodies

Cell lines, MCF-10A, MDA-MB-231 and MCF-7, were obtained from American Type Culture Collection (ATCC, Manassas, VA, USA). The cells were maintained in RPMI 1640 (Thermo Fisher Scientific, Rockford, IL, USA) supplemented with 10% fetal bovine serum (FBS) (MedSupply Partners, Atlanta, GA, USA), 1% glutamine, 100 mg/ml penicillin, and 100 mg/ml streptomycin (Life Technologies, Carlsbad, CA, USA) at 37 °C in humidified air with 5% CO_2_. The mouse monoclonal anti-Mortalin antibody (anti-Grp75) was purchased from Abcam, Inc. (Cambridge, MA, USA).

### Peptides

Our previous research showed that an extensive genetic analysis of Nef revealed several highly conserved N-terminal domains that were necessary and sufficient for Nef-induced exNef secretion. One of these domains, the novel SMR (^66^VGFPV^70^), is highly conserved across all HIV-1 clades, HIV-2, and SIV. A peptide containing the SMR sequence (SMRwt; described in^43^) was synthesized. A control peptide, SMRmut, in which valine-65 was replaced by alanine (AGFPV) was a negative control (SMRmut; described in ^43^). Both SMRwt and SMRmut peptides were custom-made by InnoPep Inc. (San Diego, CA, USA). In addition, modified forms of the SMRwt and SMRmut peptides were generated as follows: SMRwt-CPP, PEG-SMRwt-CLU, SMRmut-CPP, and PEG-SMRmut-CLU (described in^42^). CPP is a cell-penetrating peptide derived from HIV-1 tat protein (C-terminus), PEG is poly-ethylene–glycol (N-terminus), and CLU is clusterin (C-terminus).

### Breast cancer peptide uptake

MDA-MB-231 and MCF-7 breast cancer cells were treated with SMR peptides using the Chariot™ reagent (Active Motif, Carlsbad, CA, USA), as previously described^43^. Cells were collected after 24 or 48 h, and the cell pellets were resuspended with 1 × Lysis buffer for Western immunoblot analysis.

### DNA constructs

The following clones were constructed and followed the BLOCK-iT Pol II miRNAi expression vector kit (Invitrogen Corporation, Carlsbad, CA, USA) per the manufacturer’s instructions. Briefly, the HSPA9 (Mortalin) primers, Hmi 408224 to Hmi 408227^43^, and Vimentin primers, Hmi 418256 to Hmi 418259, were used to generate expression vectors miR-Mortalin and miR-Vimentin that express Mortalin and Vimentin inhibitory microRNAs (miR-Mortalin and miR-Vimentin). The kit also contained a corresponding pcDNA6.2-GW negative control plasmid (miR-neg) that produces a miRNAi predicted not to target any known vertebrate gene. This was used as a negative control.

### Transfections with miR-mortalin and miR-vimentin

MDA-MB-231 and MCF-7 breast cancer cells were transfected using Amaxa’s Nucleofector kit (Lonza Walkersville Inc., Walkersville, MD, USA) according to the manufacturer’s instructions. Briefly, the breast cancer cells were suspended in Nucleofector solution, added to miR-Mortalin or miR-Vimentin, and transferred to an Amaxa-certified cuvette for Nucleofector II apparatus application.

### Western immunoblot analysis

Western blot was performed following established methods^42^. Breast cancer cells were lysed in RIPA buffer (Sigma-Aldrich, USA). Proteins were separated using 4–20% Criterion TM TGXTM Precast Gel (Bio-Rad, USA) by electrophoresis and transferred onto the nitrocellulose membrane. (Note: Blots were cut prior to hybridization with antibodies.) The membranes were blocked with 5% dry milk in 0.1% Tween in Tris-buffered saline. The primary antibodies used for the analysis were mouse anti-human Vimentin (V9) monoclonal antibody (dilution 1:500; Santa Cruz Biotechnology, Santa Cruz, CA, USA), mouse anti-E-Cadherin (G-10) monoclonal antibody (dilution 1:500; Santa Cruz Biotechnology, Santa Cruz, CA, USA), rabbit anti-Grp-75 polyclonal (dilution 1:1000; Abcam Inc. Boston, MA, USA), mouse anti-human Alix (1A12) monoclonal antibody (dilution 1:500; Santa Cruz Biotechnology, Santa Cruz, CA, USA), and mouse anti-human Tubulin monoclonal antibody (dilution 1:2000; Sigma–Aldrich, USA). Primary antibody incubation was overnight at 4 °C. Membranes were washed thrice, and horseradish peroxidase-conjugated secondary antibody incubation (dilution 1:2000; goat anti-mouse IgG [H + L] antibody, HRP, and dilution 1:2000; goat anti-rabbit IgG [H + L] antibody, HRP, Thermo Fisher Scientific, Carlsbad, CA, USA) was for one hour at room temperature. Protein expression profiles were detected with Western blotting luminol reagent (Santa Cruz Biotechnology, Santa Cruz, CA, USA) and quantitated using Image J software (NIH, Bethesda, MD, USA).

### Cell wound-healing assay

Breast cancer cells were plated in a 96-well plate and grown for approximately 48 h to 95–100% confluency. Wounding was performed by scraping the cell monolayer with a 10 µL pipette tip. Medium and nonadherent cells were removed, followed by two washes with PBS. Next, various concentrations of SMR peptides (0–1120 nM/mL) were applied. Breast cancer cells were permitted to migrate into the clearing area for 18 h, and wound closure was monitored by microscopic examination.

### Fluorescence-based cell migration assay

Following the manufacturer's instructions, the fluorescence-based cell migration assay was performed with InnoCyte (EMD Millipore Corporation, Temecula, CA, USA)^2,3,41^. A total of 2.5 × 10^4^ breast cancer cells in the presence or absence of SMRwt peptides or Latrunculin A inhibitors were added to the upper chamber and allowed to migrate through the membrane with 8-µm pore-sizes for 24 h at 37 °C and 5% CO_2_ atmosphere. Breast cancer cells that migrated through the membrane were detached and labeled with Calcein-AM fluorescent dye. Fluorescence was measured using a Fluorescence Plate Reader (Molecular Devices. Sunnyvale, CA, USA) with an excitation wavelength of 485 nm and an emission wavelength of 515 nm.

### Co-culture and tumor cell transendothelial migration assay

The transendothelial migration of breast cancer cells was detected using CytoSelectTM (Cell Biolabs, Inc., San Diego, CA, USA) following the manufacturer’s instructions. The transwell cell culture chamber with polycarbonate filters (8 µm pore size; 0.33 cm^2^ area) This co-culture system mimics the in vivo orientation of endothelial and tumor cells during tumor cell intravasation tumor cells to make close contacts with HUVEC basal pole. 1 × 10^5^ HUVEC were seeded first on the lower side of the Transwell filter and grown to confluence for 48 h. 2 × 10^5^ of the overnight serum-starved transfected breast cancer cells were labeled with a fluorescent dye seeded to the monolayer of the HUVEC cells. The insert was then transferred to a new plate containing a fresh medium with 10% fetal bovine serum. The assay was performed at 37 °C for 24 h. non-migrating cells at the top were removed, whereas cells that migrated to the bottom of the membrane were first dissociated from the membrane, then lysed and quantified using CyQuant GR fluorescent dye 480 nm/520 nm.

### Transwell migration and invasion assays

Transwell migration and invasion assays were performed as Creative Bioarray described (https://www.creative-bioarry.com/transwell-migration-and-ivasion-assays.htm). Note: for the invasion assay, Matrigel (1 mg/mL) was added to the upper compartment of the transwell membrane inserts with 8 µm pore size polycarbonate filters (Millipore-Sigma, CA, USA) and solidified through incubation at 37 °C for 2 h. 1 × 10^6^ breast cancer cells were treated overnight with different SMR peptide dosages (in µM) as follows: 0, 0.035, 0.7, 0.14, 0.28, 0.56, 1.12, 2.24. Next, breast cancer cells (1 × 10^6^ cells per well) were resuspended in 100 µL of FBS-free media and seeded to the top of the transwell membrane inserts. RPMI 1640 media with 10% FBS was added to the lower compartment, and the plate was incubated at 37 °C for 24 h. The cells that migrated/invaded the lower surface of the membrane were fixed with 70% ethanol and stained with crystal violet for viewing using a 10 × objective under an inverted microscope.

### Exosome isolation and purification

Exosomes were isolated from breast cancer cells by established differential centrifugation and ultracentrifugation^42^. Untreated or treated SMRmut peptide breast cancer cells were used as controls. Treated and untreated breast cancer cell culture supernatants underwent sequential centrifugations as follows: 400×*g* for 10 min and 10,000×*g* for 30 min, and 200,000×*g* for 2 h. The pelleted exosomes were washed, resuspended with PBS, and stored at 4 °C until use for Nanosight analysis.

### PEG-SMRwt-CLU exosome preparation

MCF-10A cells were treated with PEG-SMRwt-CLU peptide using the Chariot™ reagent (Active Motif, Carlsbad, CA, USA), as previously described^43^. The cell culture supernatant was collected after 24 or 48 h, and exosomes containing the PEG-SMRwt-CLU peptide were harvested via ultracentrifugation.

### Nanoparticle tracking analysis measurement with Nanosight NS300

All samples were diluted in PBS (1:100) to a final volume of 1 mL. The exosomes' size and distribution were determined by the NanoSight NS300 (Malvern Panalytical Inc. Westborough MA, USA). The following settings were set according to the manufacturer’s software manual (NanoSight NS300 User Manual, MAN0541-02-EN). Five 1-min videos were captured for each measurement under the following conditions: cell temperature at 25 °C and syringe speed at 40 µL/s. After capture, the videos were analyzed by the in-build NanoSight Software NTA 3.1 Build 3.1.46 with a detection threshold of 5. Hardware: embedded laser: 45 mW at 488 nm.

### Statistical analysis

Data are expressed as the mean ± standard deviation (S.D.). Shapiro–Wilk and Kolmogorov–Smirnov tests were used to determine the normality of the data distributions. Equal variance was assessed with the Brown-Forsythe test. A nonlinear regression with the Inhibitor vs Response curve was used for SMR peptide effects on exosome release, invasion, and migration. An ANOVA with multiple comparisons was used in comparing the different modified SMR peptide effects and Mortalin’s role in migration. A *p*-value ≤ 0.05 was considered significant. These statistical analyses were performed using GraphPad Prism 9 (GraphPad Software, La Jolla, California, USA).

### Ethics approval and consent to participate

The current study was approved and reviewed by of the Institutional Review Board of the Morehouse School of Medicine.

### Consent for publication

All authors have read and approved the final manuscript for publication.

## Results

### Indirect SMRwt exosome exposure and direct treatment with SMRwt peptide inhibited breast cancer cell migration

**Figure 2:**
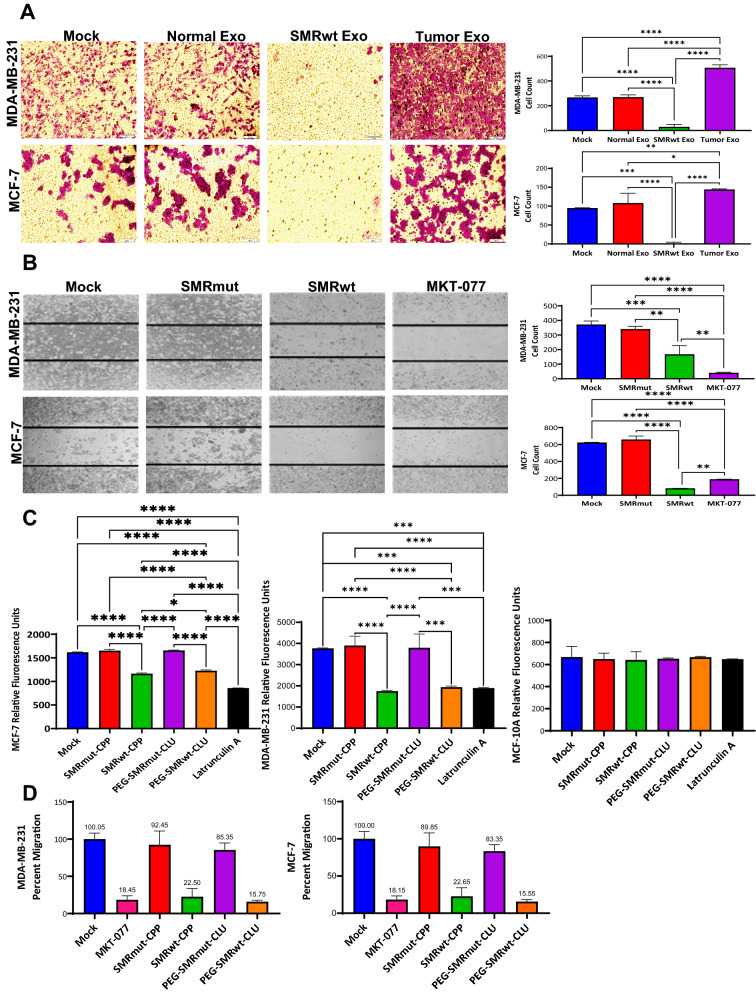
MDA-MB-231 and MCF-7 breast cancer cell migration was inhibited by exposure to SMRwt exosomes and direct treatment with SMRwt peptide. (**A**) PEG-SMRwt-CLU exosomes significantly inhibited migration of MDA-MB-231 and MCF-7 breast cancer cells, whereas tumor exosomes significantly promoted migration in these cell lines. Breast cancer cells were treated with either 6 × 10^7^ normal exosomes, tumor exosomes or SMRwt exosomes for 24 h and seeded in a transwell membrane insert. Migrated breast cancer cells were viewed underneath an inverted microscope. *p-value < 0.05, **p-value < 0.008, ***p-value = 0.0001, ****p-value < 0.0001. (**B**) Direct treatment of MCF-7 and MDA-MB-231 breast cancer cells with SMRwt peptide significantly inhibited migration in the transwell wound healing assay. Breast cancer cells were cultured for 24 h and then treated with IC50 0.56 µM (for MDA-MB-231), 1.12 µM (for MCF-7) SMRwt peptide, or 1 µM MKT-077 (control). Following 24 h, the wound healing assay was performed. **p-value = 0.001, ***p-value = 0.0004, ****p-value < 0.0001. (**C**) MDA-MB-231 and MCF-7 breast cancer cell migration was significantly blocked by all SMRwt peptides, while no effect was observed on the non-tumorigenic MCF-10A cells. Breast cancer cells were treated with IC50 0.56 µM (for MDA-MB-231), 1.12 µM (for MCF-7) SMRwt peptide or 3 µM Latrunculin A (control) for 16 h, and the Cell Migration Assay was performed (Calbiochem manual). Relative fluorescence unit of migration was determined at an excitation wavelength of 485 nm and an emission wavelength of 515 nm. *p-value < 0.05, ***p-value = 0.0001, ****p-value < 0.0001. (**D**) Breast cancer cell migration through an HUVEC monolayer was inhibited by the SMRwt peptides in a Cytoselect Tumor Transendothelial Migration Assay. Migration only at 15.75% and 15.55% following treatment with PEG-SMRwt-CLU of MDA-MB-231 and MCF-7 breast cancer cells, respectively.

PEG-SMRwt-CLU exosomes dramatically inhibited transwell migration of MDA-MB-231 and MCF-7 breast cancer cells to approximately 90% and 98% lower migrated cell counts, respectively, compared to the mock (buffer treatment), with *p*-values < 0.0001 (Fig. 2A). Conversely, breast cancer derived exosomes significantly promoted migration of MDA-MB-231 and MCF-7 breast cancer cells by approximately 90% and 52% greater migrated cell counts as compared to the mock with *p*-values < 0.0001 and < 0.008 (Fig. 2A). The mock and normal exosome-treated conditions did not significantly differ. Correspondingly, direct treatment of MDA-MB-231 and MCF-7 with SMRwt peptide inhibited transwell migration to approximately 55% and 87% lower migrated cell counts than the mock with *p*-values = 0.0004 and < 0.0001 (Fig. 2B). The SMRmut and mock conditions did not significantly differ. Regardless of the SMRwt peptide modification, both versions (SMRwt-CPP and PEG-SMRwt-CLU) significantly inhibited breast cancer cytoselect migration compared to the mock condition (Fig. 2C). Further, to confirm the inhibitory role of the SMRwt peptide in breast cancer migration, a transendothelial migration assay was performed and revealed effective inhibition by both versions of the SMRwt peptides. MDA-MB-231 breast cancer migration was reduced to 22.50% (SMRwt-CPP) and 15.75% (PEG-SMRwt-CLU) relative to the mock condition (100.05%) (Fig. 2D). MCF-7 breast cancer migration was reduced to 22.65% (SMRwt-CPP) and 15.55% (PEG-SMRwt-CLU) close to the mock condition (100%) (Fig. 2D).

### SMRwt peptide inhibited breast cancer cell migration and invasion in a dose-dependent manner

**Figure 3:**
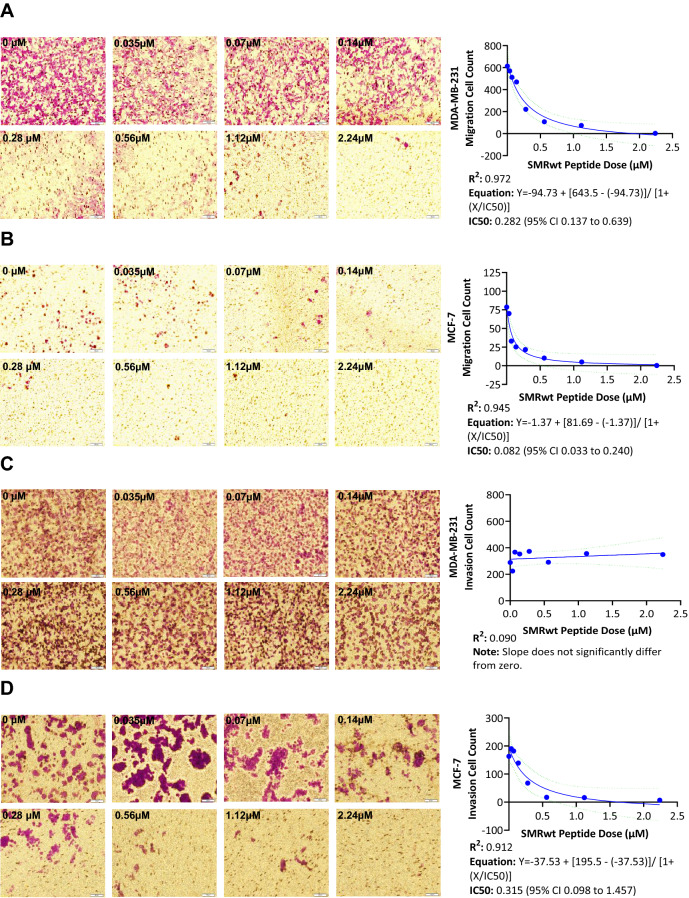
PEG-SMRwt-CLU peptide inhibited MCF-7 breast cancer cell migration and invasion as well as MDA-MB-231 breast cancer cell migration in a dose dependent manner by Transwell Migration Assay. (**A**, **B**) Breast cancer cells were incubated with PEG-SMRwt-CLU peptide at varying dosages (0–2.24 µM) at 37 °C for 24 h and seeded in a transwell membrane insert. Migrated breast cancer cells were stained and viewed underneath an inverted microscope (3 fields of view) to obtain an average migrated cell count. Nonlinear regression analysis was performed for MDA-MB-231 and MCF-7 breast cancer migrated cell counts with strong R^2^ values of 0.972 and 0.945. The calculated IC50 for migration inhibition was approximately 3.4 × greater for MDA-MB-231 at 0.282 as compared to 0.082 for MCF-7. (C-D) Similar to the migration assay, breast cancer cells were incubated with PEG-SMRwt-CLU peptide at varying dosages (0–2.24 µM) at 37 °C for 24 h and seeded in a transwell membrane insert. However, with the invasion assay, matrigel was used to simulate the ECM. Invaded breast cancer cells were stained and viewed underneath an inverted microscope (3 fields of view) to obtain an average invaded cell count. Nonlinear regression analysis was performed for MDA-MB-231 and MCF-7 breast cancer invaded cell counts. While invasion was inhibited for MCF-7 with a strong R^2^ value of 0.912 and an IC50 of 0.315, MDA-MB-231 cells were not observed to have any change in invasion capability following exposure to PEG-SMRwt-CLU peptide.

MDA-MB-231 and MCF-7 breast cancer cell migration were inhibited in a dose-dependent fashion by the SMRwt peptide with near-complete elimination of migration at the highest dosage level (2.24 µM) (Fig. 3A,B). Nonlinear regression analysis was used to determine the following equations for MDA-MB-231 and MCF-7: Y = − 94.73 + [643.5 − (− 94.73)]/[1 + (X/IC50)] with an R^2^ value of 0.972 and Y = − 1.37 + [81.69 −  (− 1.37)]/[1 + (X/IC50)] with an R^2^ value of 0.945 (Fig. 3A,B). The IC50 for MDA-MB-231 migration inhibition was 0.282 µM (95% CI 0.137–0.639) (Fig. 3A,B). The IC50 for MCF-7 migration inhibition was 0.082 µM (95% CI 0.033–0.240) (Fig. 3A,B). In contrast, the invasion assay differed between cell lines with MCF-7 invasion effectively inhibited and MDA-MB-231 invasion unaffected by the presence of the SMR peptide within the dose levels administered (Fig. 3C,D). A simple linear regression revealed that the MDA-MB-231 invasion data did not significantly differ from a zero slope, reaffirming the lack of impact SMRwt peptide had on the invasion capability (Fig. 3C). In addition, an ANOVA with multiple comparisons was performed to compare the mean MDA-MB-231 invaded cell count for the control to that of the different SMR peptide dose conditions, but only one significant difference was identified at an SMR peptide concentration of 0.28 μM (*p*-value < 0.05) (Fig. 3C). MCF-7 invasion was strongly inhibited with increasing dosages of SMRwt peptide. Like the migration assay for MCF-7, the invasion assay had near elimination of invasion at the highest dosage level (2.24 µM) (Fig. 3D). Nonlinear regression analysis for MCF-7 analysis determined the following equation: Y = − 37.53 + [195.5 −  (− 37.53)]/[1 + (X/IC50)] with an R^2^ value of 0.912 (Fig. 3D). The IC50 for MCF-7 invasion inhibition was 0.315 µM (95% CI 0.098–1.457) (Fig. 3D). In addition, an ANOVA with multiple comparisons was performed to compare the mean MCF-7 invaded cell count for the control to that of the different SMR peptide dose conditions. Significant differences were identified at the following SMR peptide concentrations (in μM) 0.28 (*p*-value < 0.005), 0.56 (*p*-value < 0.0005), 1.12 (*p*-value < 0.00005), and 2.24 (*p*-value < 0.00005) (Fig. 3D). As the invasion assay for MCF-7 required a higher dosage to attain inhibition with the SMRwt peptide than the migration assay, the MDA-MB-231 cells may have invasion be inhibited at high SMRwt peptide dosages.

### Mortalin is key to breast cancer cell migration

**Figure 4:**
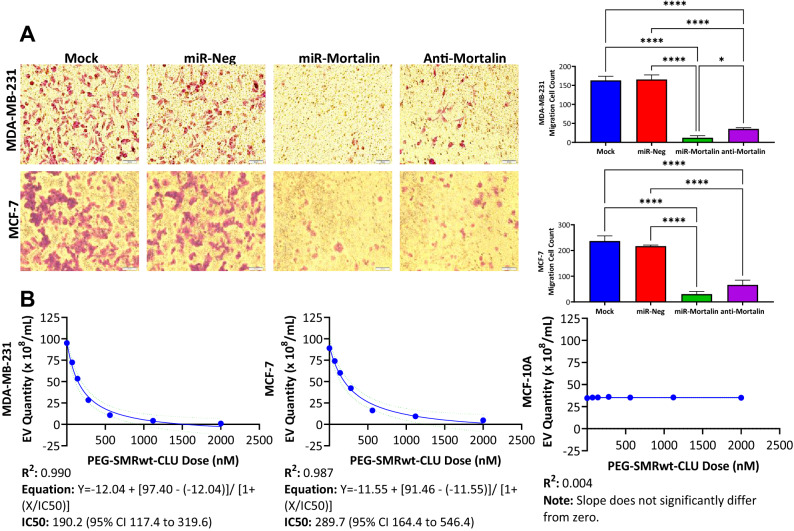
Mortalin is involved in MDA-MB-231 and MCF-7 breast cancer cell migration and PEG-SMRwt-CLU blocks breast cancer exosome release. (**A**) Breast cancer cells were transfected with either miR-Negative, miR-Mortalin, or anti-Mortalin antibody and then incubated at 37 °C for 24 h. After 24 h, breast cancer cells were collected and 1 × 10^5^ cells/well were used for a Transwell migration assay. Migrated breast cancer cells were stained and viewed underneath an inverted microscope (3 fields of view) to obtain an average migrated cell count. miR-Mortalin and anti-mortalin treated breast cancer cells had significantly reduced migration. *p-value < 0.05, ****p-value < 0.0001 (**B**) Breast cancer cells were treated with varying dosages of PEG-SMRwt-CLU peptide (0–2000 nM) and incubated at 37 °C for 48 h. Nanoparticle-tracking analysis (NTA) was performed to characterize the EVs isolated from the conditioned media. MDA-MB-231 and MCF-7 exosome release were blocked by PEG-SMRwt-CLU peptide treatment with strong R^2^ values of 0.990 and 0.987. Non-tumorigenic MCF-10A epithelial cell exosome release was unaffected by application of the PEG-SMRwt-CLU peptide.

To investigate the role of mortalin in breast cancer migration, breast cancer cells were transfected with either miR-Negative, miR-Mortalin, or exposed to anti-Mortalin antibody and assessed for transwell migration capability. miR-Mortalin and anti-Mortalin treated cells had significantly reduced migration compared to the mock condition (Fig. 4A). Specifically, for MDA-MB-231 miR-Mortalin treated cell migration was lowered 92.6%, and anti-Mortalin treated cell migration was lowered 78.3% as compared to the mock condition (Fig. 4A). For MCF-7 miR-Mortalin treated cell migration was reduced 87.3%, and anti-Mortalin treated cell migration was lowered 72.1% compared to the mock condition (Fig. 4A). The mock and miR-Negative conditions did not significantly differ. Also, the miR-Mortalin and anti-Mortalin conditions did not significantly differ.

### SMRwt peptide blocks breast cancer exosome release but has no effect on non-tumorigenic MCF-10A epithelial cells

MDA-MB-231 and MCF-7 exosome release were blocked by SMRwt peptide treatment. Nonlinear regression analysis determined the following equations for MDA-MB-231 and MCF-7: Y = − 12.04 + [97.40 −  (− 12.04)]/[1 + (X/IC50)] with an R^2^ value of 0.990 and Y = − 11.55 + [91.46 − (− 11.55)]/ [1 + (X/IC50)] with an R^2^ value of 0.987 (Fig. 4B). The IC50 for MDA-MB-231 SMRwt exosome blockade was 190.2 nM (95% CI 117.4–319.6) (Fig. 4B). The IC50 for MCF-7 SMRwt exosome blockade was 289.7 nM (95% CI 164.4–546.4) (Fig. 4B). Interestingly, the SMRwt peptide had no impact on exosome release of non-tumorigenic MCF-10A epithelial cells (Fig. 4B). A simple linear regression showed that SMRwt treated MCF-10A cell exosome release data did not significantly differ from a slope of zero reaffirming the lack of impact SMRwt peptide had on blocking MCF-10A exosome release. The exact exosome release quantities are as follows: For MDA-MB-231 cells, untreated controls contained 95.07 × 10^8^ particles/mL, 70 nM contained 72.58 × 10^8^ particles/mL, 140 nM contained 51.96 × 10^8^ particles/mL, 280 nM contained 28.52 × 10^8^ particles/mL, 560 nM contained 14.44 × 10^8^ particles/mL, 1120 nM contained 4.12 × 10^8^ particles/mL and 2000 nM contained 2.15 × 10^8^ particles/mL. For MCF-7 cells, untreated control contained 89.20 × 10^8^ particles/mL, 70 nM contained 73.94 × 10^8^ particles/mL, 140 nM contained 61.54 × 10^8^ particles/mL, 280 nM contained 42.29 × 10^8^ particles/mL, 560 nM contained 18.43 × 10^8^ particles/mL, 1120 nM contained 9.44 × 10^8^ particles/mL and 2000 nM contained 4.0 × 10^8^ particles/mL. NTA estimated the exosomes size to be in the range of 30–51 nm for all breast cancer cell cultures.

### Protein expression of Mortalin and Vimentin was reduced following SMRwt treatment in breast cancer cells and breast cancer derived exosomes

**Figure 5:**
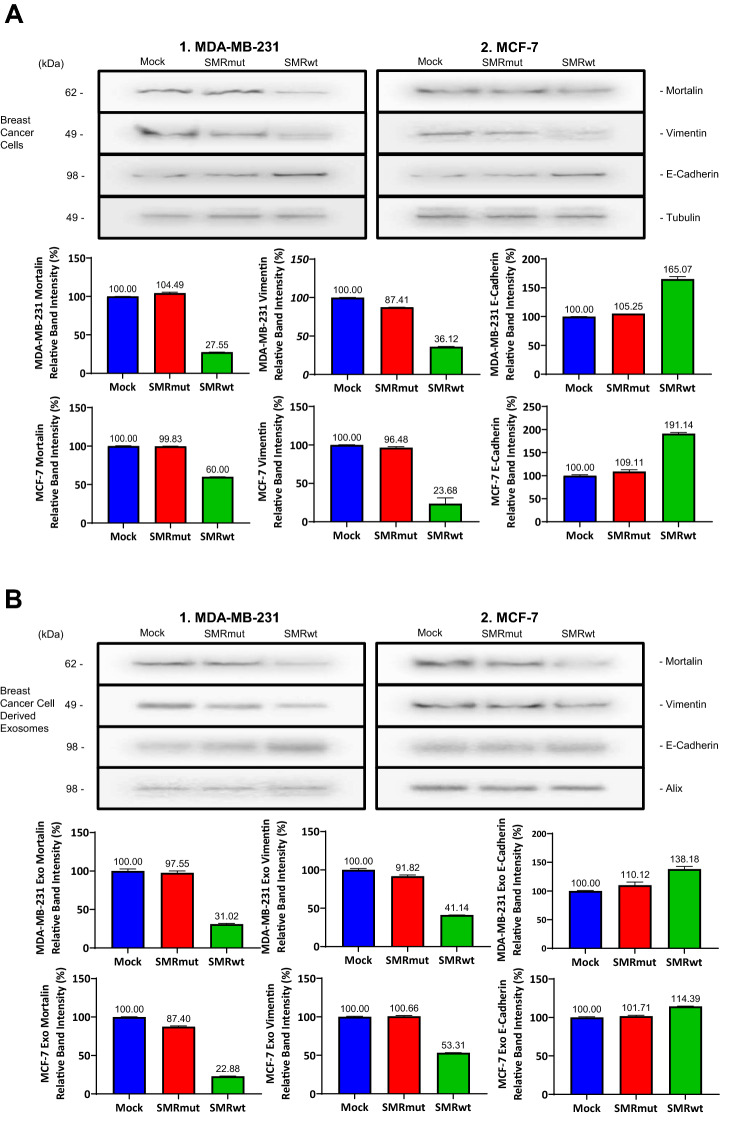
Protein expression of Mortalin and Vimentin are reduced by PEG-SMRwt-CLU inhibition in breast cancer cells and breast cancer derived exosomes. (**A**) Western blot densitometry analysis of protein expression levels of Mortalin, Vimentin, and E-Cadherin in MDA-MB-231 and MCF-7 breast cancer cells under the following conditions: Mock, SMRmut, SMRwt. Treatment with SMRwt of MDA-MB-231 and MCF-7 breast cancer cells lowered the protein expression of Mortalin to 27.66% and 60% as well as Vimentin to 14.12% and 23.68%. In addition, E-cadherin protein expression was increased in MDA-MB-231 and MCF-7 breast cancer cells to 165.07% and 191.14% exposed to SMRwt peptide. (**B**) Western blot densitometry analysis of exosome protein expression levels of Mortalin, Vimentin, and E-Cadherin in MDA-MB-231 and MCF-7 breast cancer cells under the following conditions: Mock, SMRmut, SMRwt. Treatment with SMRwt of MDA-MB-231 and MCF-7 breast cancer cells resulted in lowered exosome protein expression of Mortalin to 31.02% and 22.88% as well as Vimentin to 41.14% and 53.31%. In addition, E-cadherin protein expression was increased in MDA-MB-231 and MCF-7 breast cancer cells to 138.18% and 114.39% exposed to SMRwt peptide. Original blots/gels are presented in Supplementary Figs. 5A-1 through 5B-2.

Mortalin and Vimentin were lower for the SMRwt peptide application than SMRmut peptide or mock in MDA-MB-231 and MCF-7 cells (Fig. 5A). Treatment with SMRwt of MDA-MB-231 and MCF-7 breast cancer cells lowered the protein expression of Mortalin to 27.66% and 60% as well as Vimentin to 14.12% and 23.68% (Fig. 5A). In addition, E-cadherin protein expression was increased in MDA-MB-231 and MCF-7 breast cancer cells to 165.07% and 191.14% exposed to SMRwt peptide (Fig. 5A). These effects aligned with breast cancer derived exosome protein expression following SMRwt exposure with reduced Mortalin and Vimentin and increased E-cadherin (Fig. 5B). Treatment with SMRwt of MDA-MB-231 and MCF-7 breast cancer cells resulted in lowered exosome protein expression of Mortalin to 31.02% and 22.88% and Vimentin to 41.14% and 53.31% (Fig. 5B). In addition, E-cadherin protein expression was increased in MDA-MB-231 and MCF-7 breast cancer cells to 138.18% and 114.39% exposed to SMRwt peptide (Fig. 5B).

### Knockdown of mortalin and vimentin effects on breast cancer cells parallel SMRwt treatment for cellular and exosome protein expression profiles

**Figure 6:**
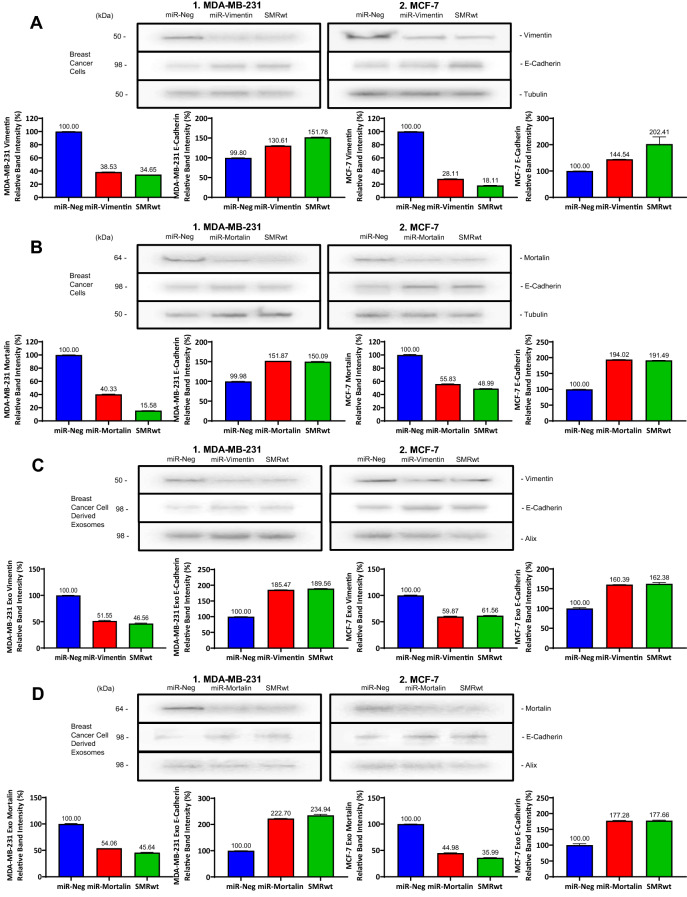
Treatment with PEG-SMRwt-CLU peptide paralleled the effects of knockdown of Mortalin and Vimentin with reduced protein expression observed in breast cancer cells and breast cancer derived exosomes. (**A**, **B**) Breast cancer cells were transfected with either miR-Mortalin, miR-Vimentin, miR-negative (negative control), or treated with SMRwt peptide. Western blot densitometry analysis was used to assess protein expression levels of Mortalin, Vimentin, and E-Cadherin. SMRwt treatment and knockdown of Mortalin and Vimentin had similar effects on Mortalin, Vimentin, and E-Cadherin protein expression levels. Specifically, protein levels of Mortalin and Vimentin were reduced, and E-Cadherin was increased. (**C**, **D**) Breast cancer cells were transfected with either miR-Mortalin, miR-Vimentin, miR-negative (negative control), or treated with SMRwt peptide. Western blot densitometry analysis was used to assess exosome protein expression levels of Mortalin, Vimentin, and E-Cadherin. SMRwt treatment and knockdown of Mortalin and Vimentin had similar effects on Mortalin, Vimentin, and E-Cadherin exosome protein expression levels. Specifically, exosome protein levels of Mortalin and Vimentin were reduced, and E-Cadherin was increased. Original blots/gels are presented in Supplementary Figs. 6A-1 through 6D-2.

To investigate SMRwt peptide effects comparatively to Mortalin and Vimentin’s role in tumor cell progression, we transfected miRNA Mortalin and miRNA Vimentin to MDA-MB-231 and MCF-7 cells and monitored for EMT. SMRwt peptide decreased Mortalin and Vimentin protein levels and increased E-cadherin levels in MDA-MB-231 and MCF-7 breast cancer cells. These effects were similar to those observed from knockdown of Mortalin and Vimentin. SMRwt treatment of MDA-MB-231 cells had protein expression levels reduced to the following: 34.65% Vimentin and 15.58% Mortalin (Fig. 6A,B). Correspondingly, miR-Vimentin and miR-Mortalin MDA-MB-231 cells had protein expression levels reduced to the following: 38.53% Vimentin and 40.33% Mortalin (Fig. 6A,B). SMRwt treatment of MCF-7 cells had protein expression levels reduced to the following: 18.11% Vimentin and 48.99% Mortalin (Fig. 6A,B). Similarly, miR-Vimentin and miR-Mortalin MCF-7 cells had protein expression levels reduced to the following: 28.11% and 55.83% (Fig. 6A,B). For MDA-MB-231 and MCF-7 cells, E-cadherin protein levels were heightened > 100% for miR-Vimentin, miR-Mortalin, and SMRwt treatment groups (Fig. 6A,B). Breast cancer exosome protein expression levels of Mortalin and Vimentin mirrored the effects observed in the parental breast cancer cells treated with SMRwt, miR-Vimentin, and miR-Mortalin (Fig. 6C,D). SMRwt breast cancer derived exosomes had Vimentin and Mortalin protein expression levels reduced to 46.56% and 45.64% for MDA-MB-231 and 61.56% and 35.99% for MCF-7 (Fig. 6C,D). Further, miR-Vimentin and miR-Mortalin breast cancer derived exosomes had Vimentin and Mortalin protein expression levels reduced to approximately 50% for MDA-MB-231 and MCF-7 (Fig. 6C,D). Breast cancer derived exosomes E-cadherin protein levels were heightened > 100% for SMRwt, miR-Vimentin, and miR-Mortalin (Fig. 6C,D). Taken together, SMRwt treatment and knockdown of Mortalin and Vimentin had similar effects on Mortalin, Vimentin, and E-Cadherin cellular and exosome protein expression levels. Specifically, cellular and exosome protein levels of Mortalin and Vimentin were reduced, and E-Cadherin was increased.

## Conclusions

Previous research in our laboratory demonstrated that the SMR region of HIV-1 Nef interacts with the cellular proteins mortalin and vimentin (Refs). These proteins play important roles in viral infection in cell growth and are involved in metastasis. Importantly, interactions of mortalin with these cellular partners can be disrupted by flooding cells with a peptide containing the SMR sequence (SMRwt). Thus, we wished to investigate the SMRwt peptide’s impact on breast cancer metastatic potential. This study investigated the effect of antagonist SMRwt peptide on MCF-7 and MDA-MB-231 tumor exosome release, migration, and invasion, as well as exosome proteomic profiling. Our results indicate: (i) exosomes promote tumor cell migration; (ii) Mortalin and Vimentin are involved in tumor migration; and (iii) SMRwt peptide inhibits breast cancer cells’ migration and invasion. These results reinforce the therapeutic value of targeting breast cancer exosome release and reinforce Mortalin and Vimentin as critical regulators and therapeutic targets in breast cancer cell progression, EMT, and metastatic potential. A greater understanding of the SMR peptide mechanism of action will benefit the therapeutic design of anti-metastatic agents.

## Discussion

Tumor cell acquisition of a migratory mesenchymal phenotype and invasion into the surrounding matrix are early events of metastasis occurrence. Proteins, such as Mortalin and Vimentin, which contribute to this early pathological migration and invasion process may be critical molecular targets for early breast cancer metastasis intervention. Recent reports indicated a correlation between Mortalin expression level with metastatic potential and tumor recurrence, suggesting the clinical application of Mortalin as a chemotherapeutic drug target. Mortalin expression levels correlate with the development of epithelial-mesenchymal-transformation (EMT), a crucial step for metastasis and inactivation of tumor suppressor p53 protein, deregulation of apoptosis, and activation of EMT signaling^31^. High levels of Mortalin in breast cancer are associated with the mesenchymal markers, whereas the epithelial markers are downregulated^44^. Malignant cells undergoing EMT acquired migration and drug resistance characteristics^31,45^. EMT is a process characterized by: (a) the absence of polarity and intercellular adhesion of epithelial cells, (b) the acquisition of mesenchyme features associated with higher motility, and (c) the altered expression of EMT biomarkers (down-regulation of epithelial marker E-cadherin and up-regulation of the mesenchymal marker Vimentin). Mortalin is upregulated in human breast cancer cells. Its depletion robustly induces cell death and growth arrest in breast cancer cell lines in culture and can interact with p53, affecting the cell cycle and survival^46,47^. Anti-tumor peptides, such as the SMRwt peptide, also induce tumor cell arrest at cell cycle G2/M in MDA-MB-231 and MCF-7 breast cancer cells^42^. In addition, the regulation of invasive and migratory properties for carcinomas involves many factors that contribute to complex intercellular crosstalk and networking^35,48^. An autocrine loop exists for the vascular endothelial growth factor (VEGF) to induce cell migration and invasion of breast cancer cells. MCF-7 cells express lower levels of VEGF than MDA-MB-231 cells, which have high invasive and migration capacities^49^. Without estrogen supplementation, MCF-7 cells do not induce metastasis in mice and have a low migration capacity in vitro^50^. Youngs et al.^51^ demonstrated a dose-dependent migratory response of breast cancer cells to increasing concentrations of exogenous CCL2. The Tumor cell acquisition of a migratory mesenchymal phenotype and invasion into the surrounding CCL2 expression has been shown to correlate with progression in pancreatic cancer^52^ and breast cancer^53^.

This study evaluated whether SMRwt peptide inhibition of breast cancer cell migration and invasion could effectively inhibit the growth of human breast cancer cells and whether the anti-tumor effect was linked to the reduction of tumor exosomes secretion. The results indicate that breast cancer cells treated with the SMRwt peptide displayed decreased function of the human chaperone protein Mortalin, significantly affecting tumor cell proliferation and reducing tumor cell invasion and migration. These results further support the role of Mortalin in breast cancer progression and support the role of the SMRwt peptide as an antagonist to Mortalin function. The SMRwt peptide also antagonizes EV release. The SMRwt peptide reduces the expression of Mortalin, which could play a role in breast cancer cell invasion and metastasis, thus suggesting potential peptide applications in early-stage breast cancer chemotherapy. This study has also demonstrated that the SMRwt peptide plays a novel and crucial role in breast cancer metastasis migration and invasion. Knockdown of Mortalin and Vimentin leads to E-cadherin expression changes, which facilitate adhesion formation and reduce Vimentin, a critical transcription factor in EMT^54^. Furthermore, present data suggest that SMRwt peptide could block breast cancer metastasis through mechanisms involving target protein Mortalin and Vimentin that decrease proliferation, tumorigenesis, and tumor exosome release. Therefore, SMRwt peptide could be a potential novel strategy for breast cancer therapy, especially in early-stage breast cancer patients.

## Supplementary Information

Supplementary Figures.

## Data Availability

All data generated or analyzed during this study are included in this published article and its supplementary information files.

## Abbreviations

SMR: Secretion modification region
EMT: Epithelial–mesenchymal transition
MET: Mesenchymal–epithelial transition
EV: Extracellular vesicles
