# Supplementary material for: Novel secretion modification region (SMR) peptide exhibits anti-metastatic properties in human breast cancer cells
Source: Sci Rep. 2022 Aug 1;12:13204. doi: 10.1038/s41598-022-17534-z (PMC9343421; doi:10.1038/s41598-022-17534-z)
Supplement: Supplementary file 1 — Supplementary Figures. [file 41598_2022_17534_MOESM1_ESM.pptx]

## Slide 1
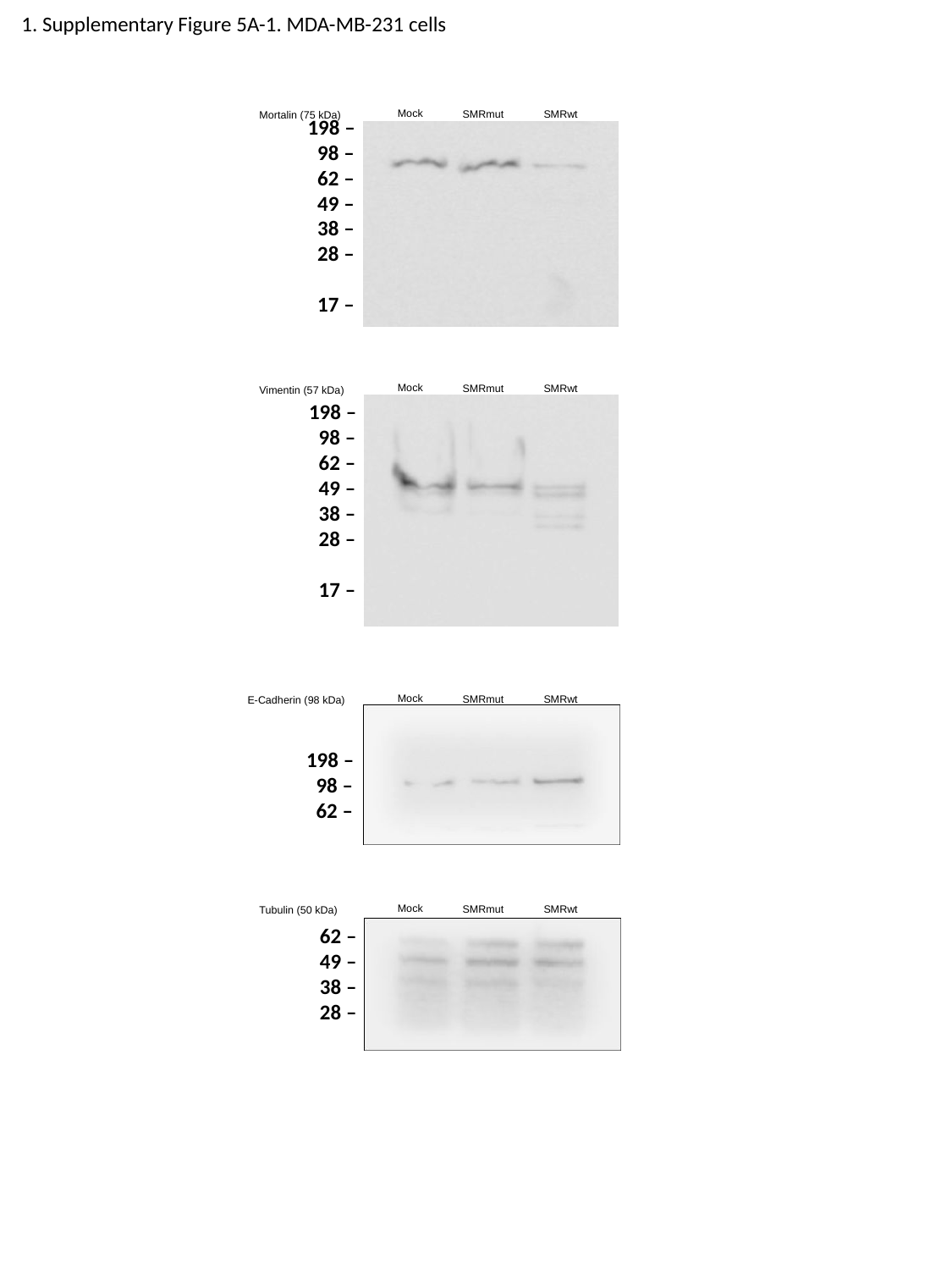

1. Supplementary Figure 5A-1. MDA-MB-231 cells
Mock
SMRmut
SMRwt
Mortalin (75 kDa)
198 –
 98 –
 62 –
 49 –
 38 –
 28 –
 17 –
Mock
SMRmut
SMRwt
Vimentin (57 kDa)
198 –
 98 –
 62 –
 49 –
 38 –
 28 –
 17 –
Mock
SMRmut
SMRwt
E-Cadherin (98 kDa)
198 –
 98 –
 62 –
Mock
SMRmut
SMRwt
Tubulin (50 kDa)
 62 –
 49 –
 38 –
 28 –

## Slide 2
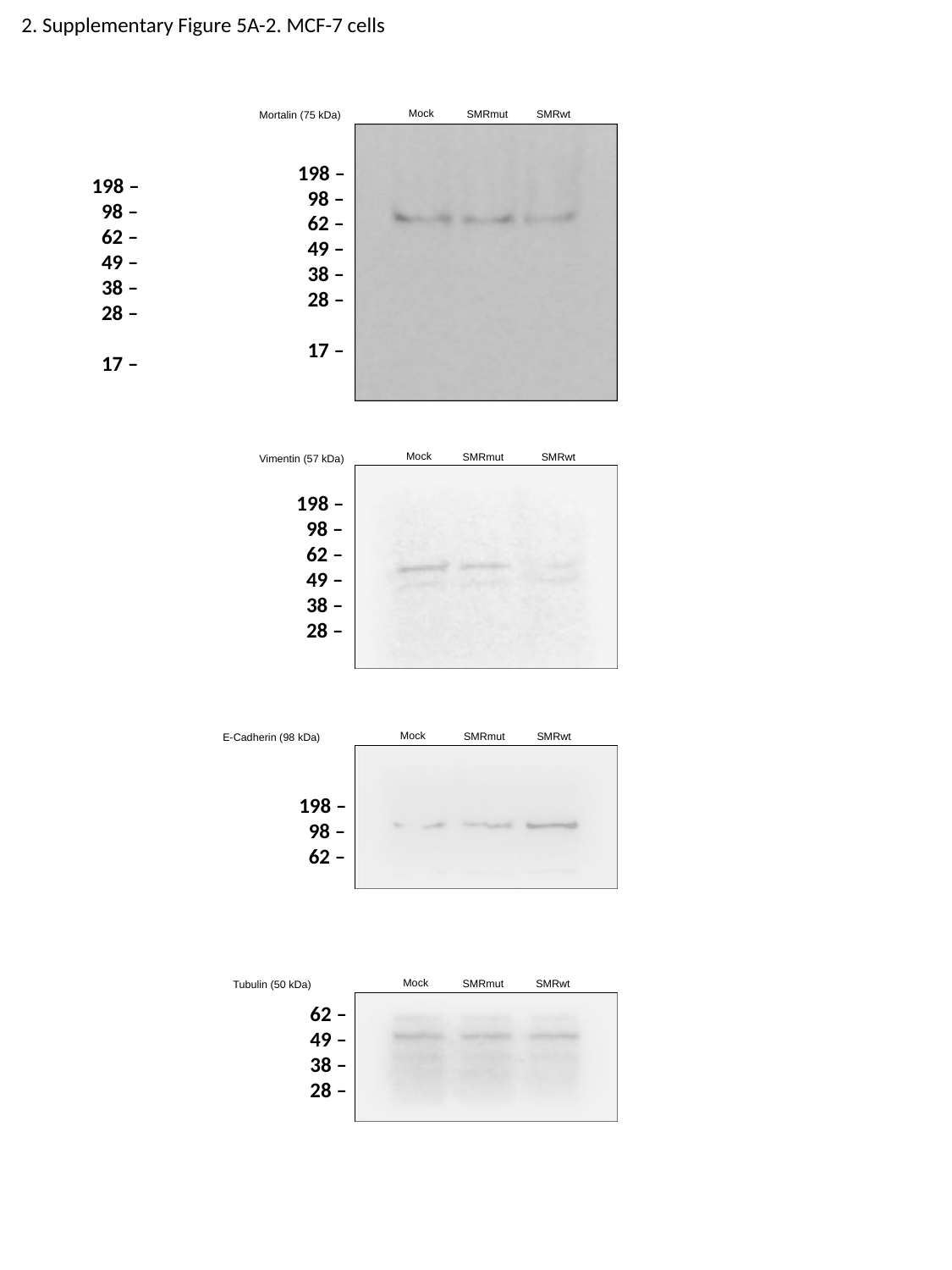

2. Supplementary Figure 5A-2. MCF-7 cells
Mock
SMRmut
SMRwt
Mortalin (75 kDa)
198 –
 98 –
 62 –
 49 –
 38 –
 28 –
 17 –
198 –
 98 –
 62 –
 49 –
 38 –
 28 –
 17 –
Mock
SMRmut
SMRwt
Vimentin (57 kDa)
198 –
 98 –
 62 –
 49 –
 38 –
 28 –
Mock
SMRmut
SMRwt
E-Cadherin (98 kDa)
198 –
 98 –
 62 –
Mock
SMRmut
SMRwt
Tubulin (50 kDa)
 62 –
 49 –
 38 –
 28 –

## Slide 3
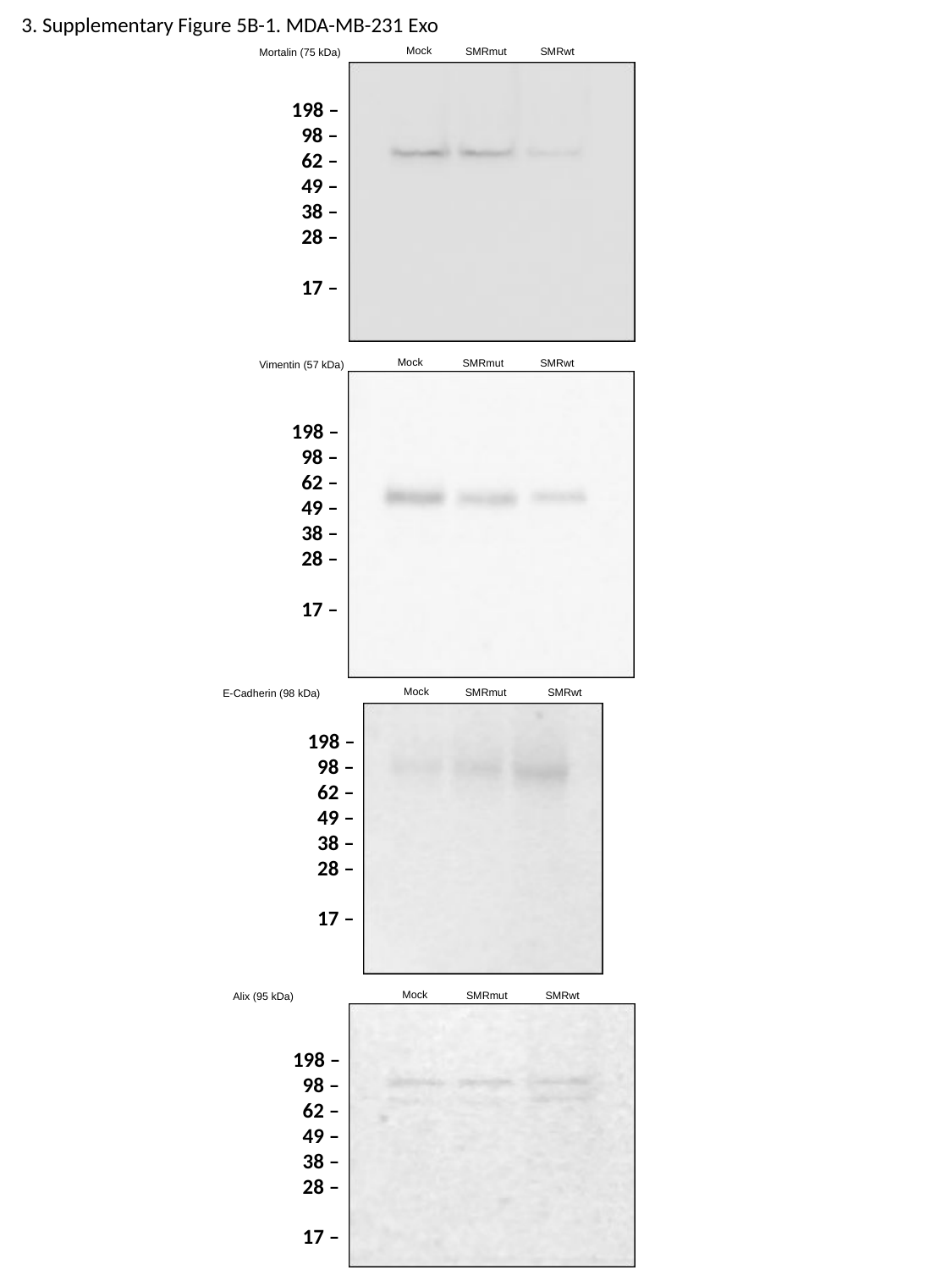

3. Supplementary Figure 5B-1. MDA-MB-231 Exo
Mock
SMRmut
SMRwt
Mortalin (75 kDa)
198 –
 98 –
 62 –
 49 –
 38 –
 28 –
 17 –
Mock
SMRmut
SMRwt
Vimentin (57 kDa)
198 –
 98 –
 62 –
 49 –
 38 –
 28 –
 17 –
Mock
SMRmut
SMRwt
E-Cadherin (98 kDa)
198 –
 98 –
 62 –
 49 –
 38 –
 28 –
 17 –
Mock
SMRmut
SMRwt
Alix (95 kDa)
198 –
 98 –
 62 –
 49 –
 38 –
 28 –
 17 –

## Slide 4
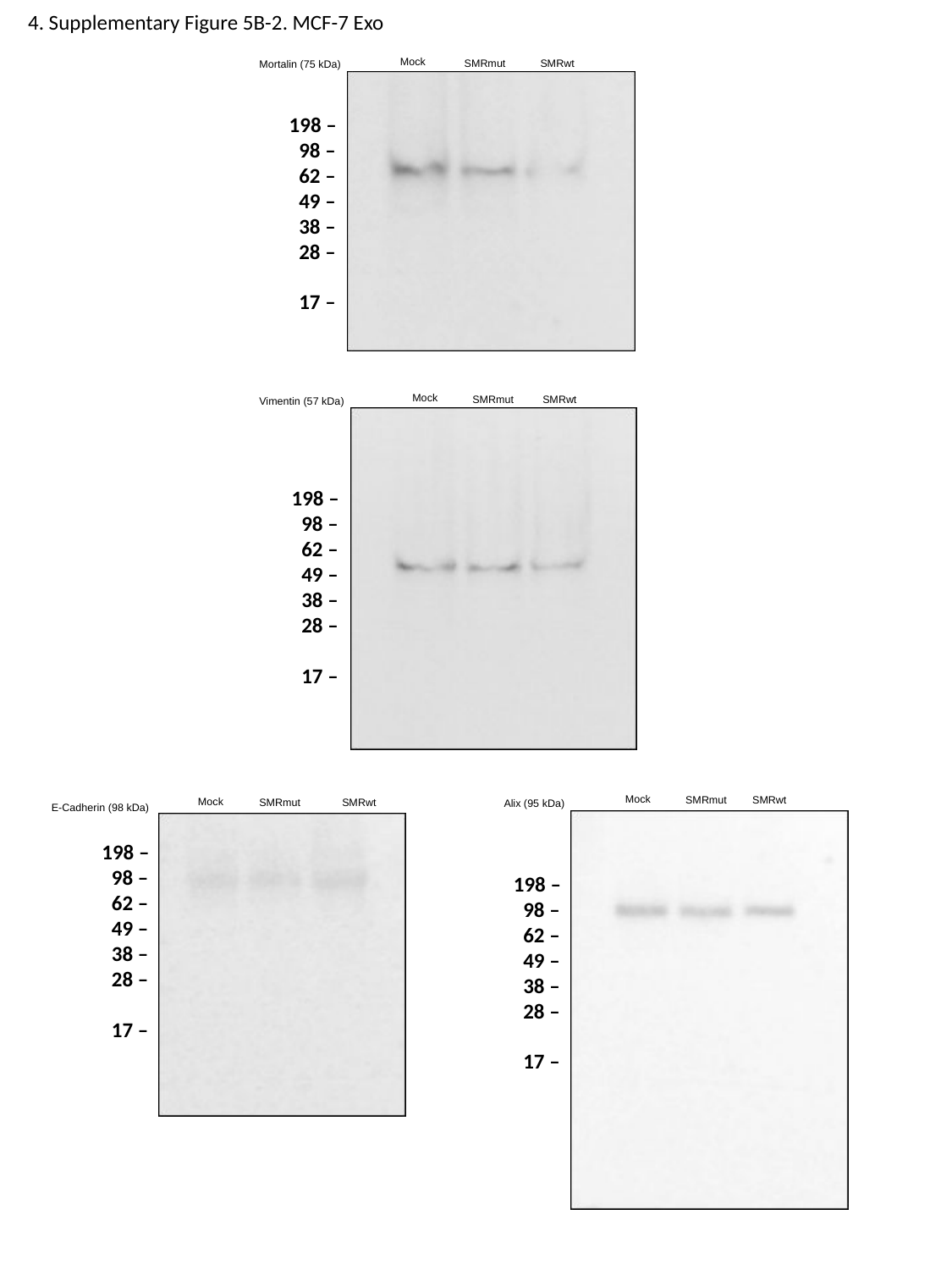

4. Supplementary Figure 5B-2. MCF-7 Exo
Mock
SMRmut
SMRwt
Mortalin (75 kDa)
198 –
 98 –
 62 –
 49 –
 38 –
 28 –
 17 –
Mock
SMRmut
SMRwt
Vimentin (57 kDa)
198 –
 98 –
 62 –
 49 –
 38 –
 28 –
 17 –
Mock
SMRmut
SMRwt
Mock
SMRmut
SMRwt
Alix (95 kDa)
E-Cadherin (98 kDa)
198 –
 98 –
 62 –
 49 –
 38 –
 28 –
 17 –
198 –
 98 –
 62 –
 49 –
 38 –
 28 –
 17 –

## Slide 5
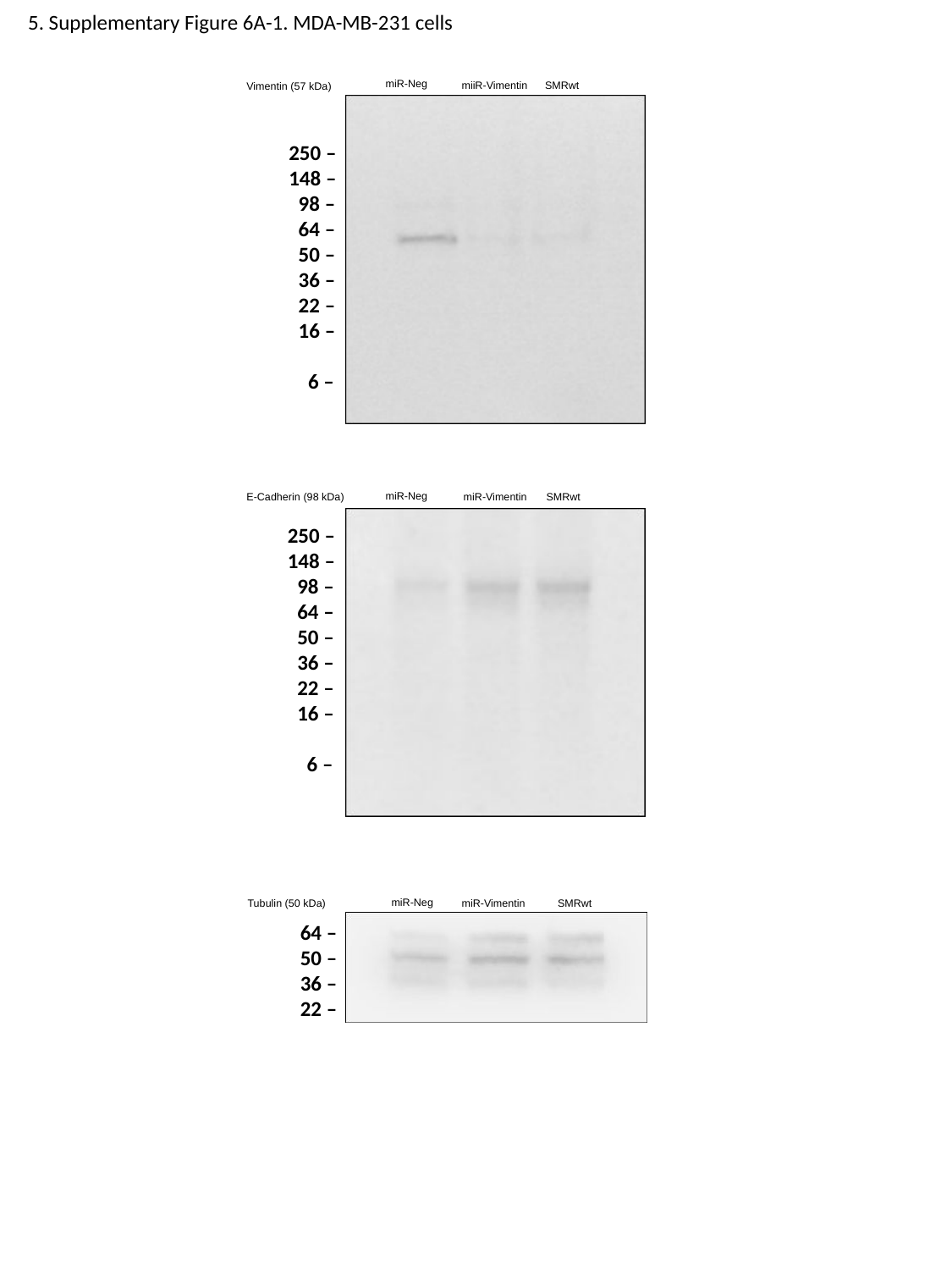

5. Supplementary Figure 6A-1. MDA-MB-231 cells
miR-Neg
miiR-Vimentin
SMRwt
Vimentin (57 kDa)
250 –
148 –
 98 –
 64 –
 50 –
 36 –
 22 –
 16 –
 6 –
miR-Neg
E-Cadherin (98 kDa)
miR-Vimentin
SMRwt
250 –
148 –
 98 –
 64 –
 50 –
 36 –
 22 –
 16 –
 6 –
miR-Neg
Tubulin (50 kDa)
miR-Vimentin
SMRwt
 64 –
 50 –
 36 –
 22 –

## Slide 6
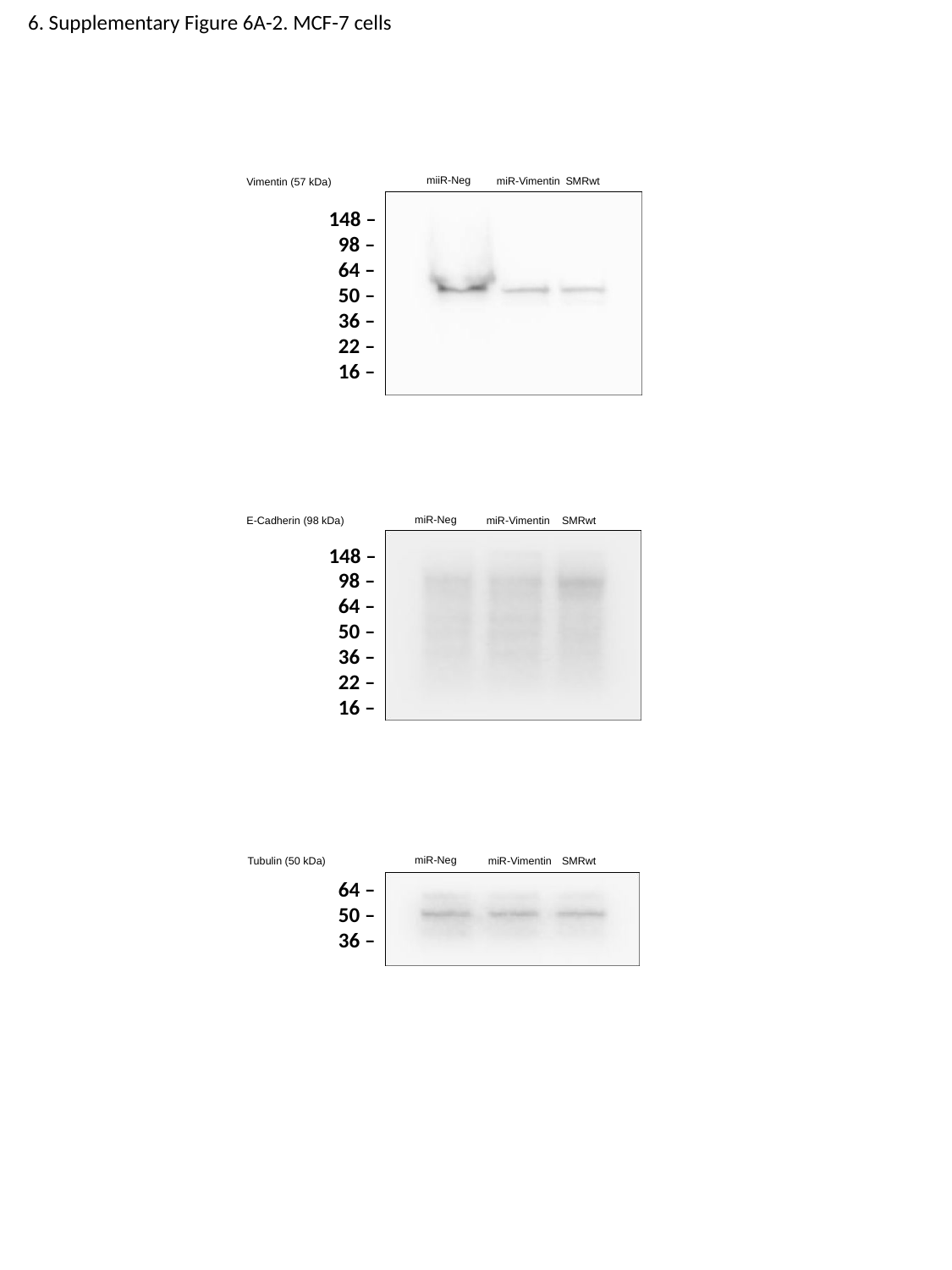

6. Supplementary Figure 6A-2. MCF-7 cells
miiR-Neg
miR-Vimentin
SMRwt
Vimentin (57 kDa)
148 –
 98 –
 64 –
 50 –
 36 –
 22 –
 16 –
miR-Neg
E-Cadherin (98 kDa)
miR-Vimentin
SMRwt
148 –
 98 –
 64 –
 50 –
 36 –
 22 –
 16 –
miR-Neg
Tubulin (50 kDa)
miR-Vimentin
SMRwt
 64 –
 50 –
 36 –

## Slide 7
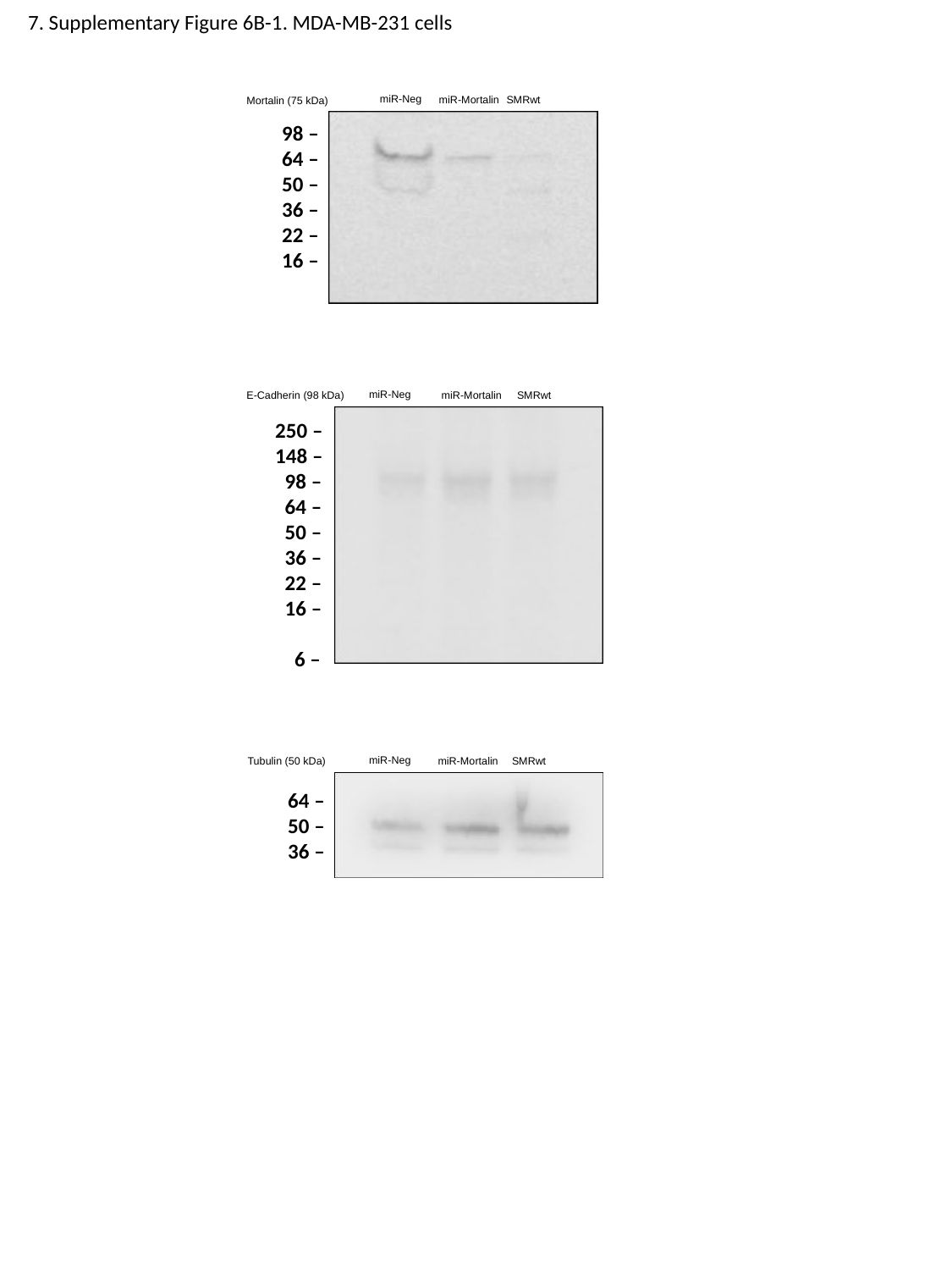

7. Supplementary Figure 6B-1. MDA-MB-231 cells
miR-Neg
miR-Mortalin
SMRwt
Mortalin (75 kDa)
 98 –
 64 –
 50 –
 36 –
 22 –
 16 –
miR-Neg
E-Cadherin (98 kDa)
miR-Mortalin
SMRwt
250 –
148 –
 98 –
 64 –
 50 –
 36 –
 22 –
 16 –
 6 –
miR-Neg
Tubulin (50 kDa)
miR-Mortalin
SMRwt
 64 –
 50 –
 36 –

## Slide 8
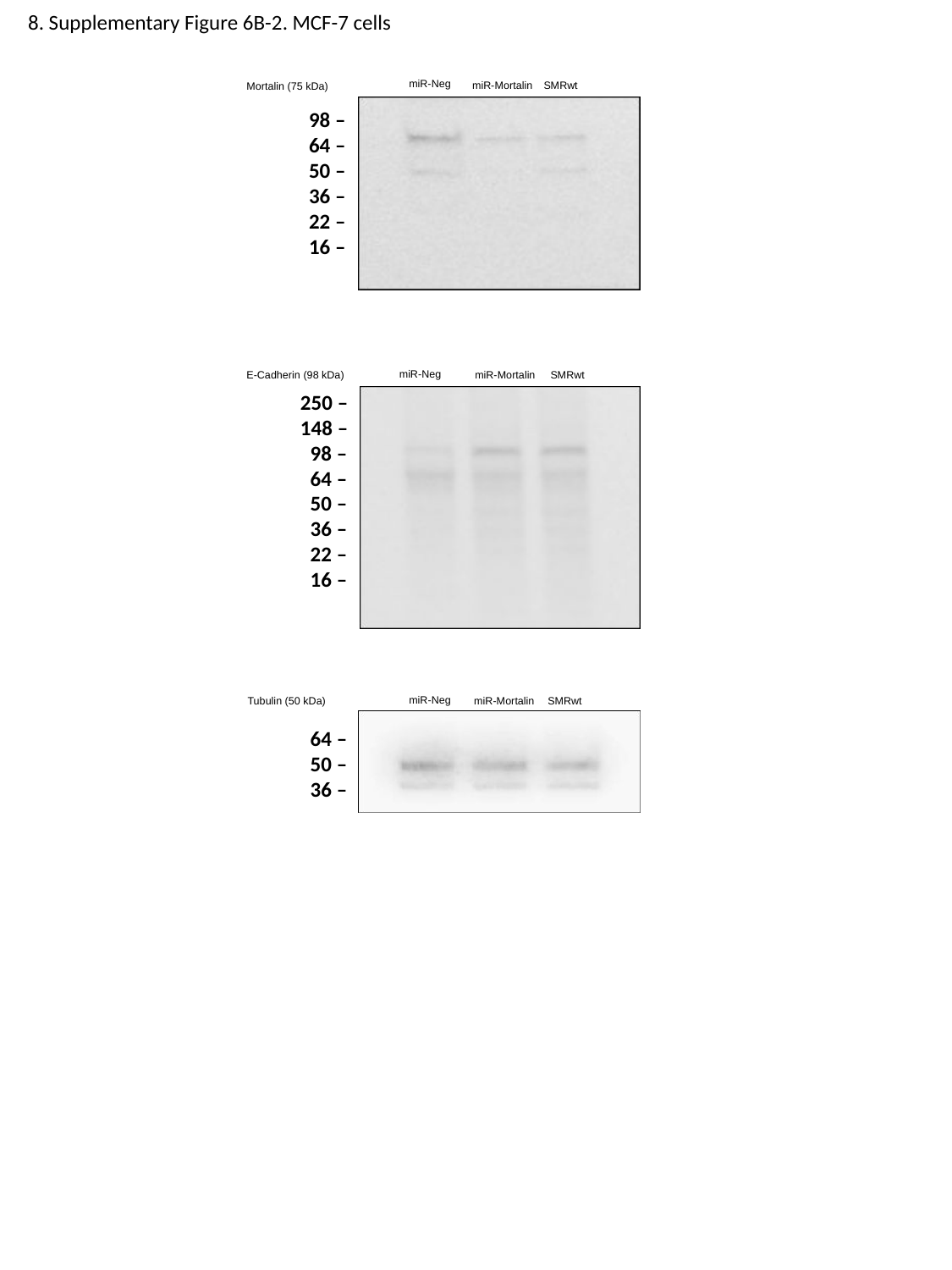

8. Supplementary Figure 6B-2. MCF-7 cells
miR-Neg
miR-Mortalin
SMRwt
Mortalin (75 kDa)
 98 –
 64 –
 50 –
 36 –
 22 –
 16 –
miR-Neg
E-Cadherin (98 kDa)
miR-Mortalin
SMRwt
250 –
148 –
 98 –
 64 –
 50 –
 36 –
 22 –
 16 –
miR-Neg
Tubulin (50 kDa)
miR-Mortalin
SMRwt
 64 –
 50 –
 36 –

## Slide 9
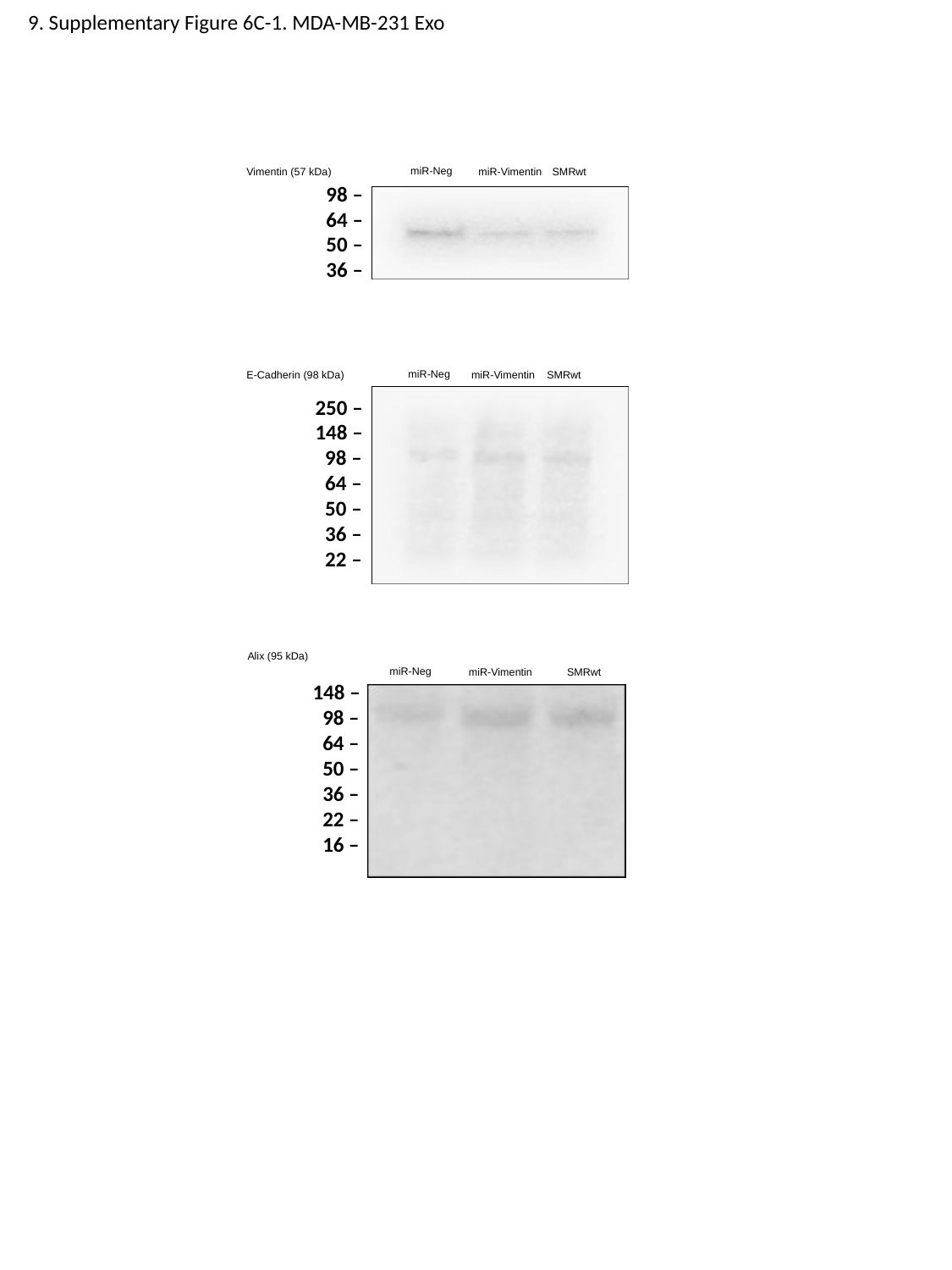

9. Supplementary Figure 6C-1. MDA-MB-231 Exo
miR-Neg
miR-Vimentin
SMRwt
Vimentin (57 kDa)
 98 –
 64 –
 50 –
 36 –
miR-Neg
E-Cadherin (98 kDa)
miR-Vimentin
SMRwt
250 –
148 –
 98 –
 64 –
 50 –
 36 –
 22 –
Alix (95 kDa)
miR-Neg
miR-Vimentin
SMRwt
148 –
 98 –
 64 –
 50 –
 36 –
 22 –
 16 –

## Slide 10
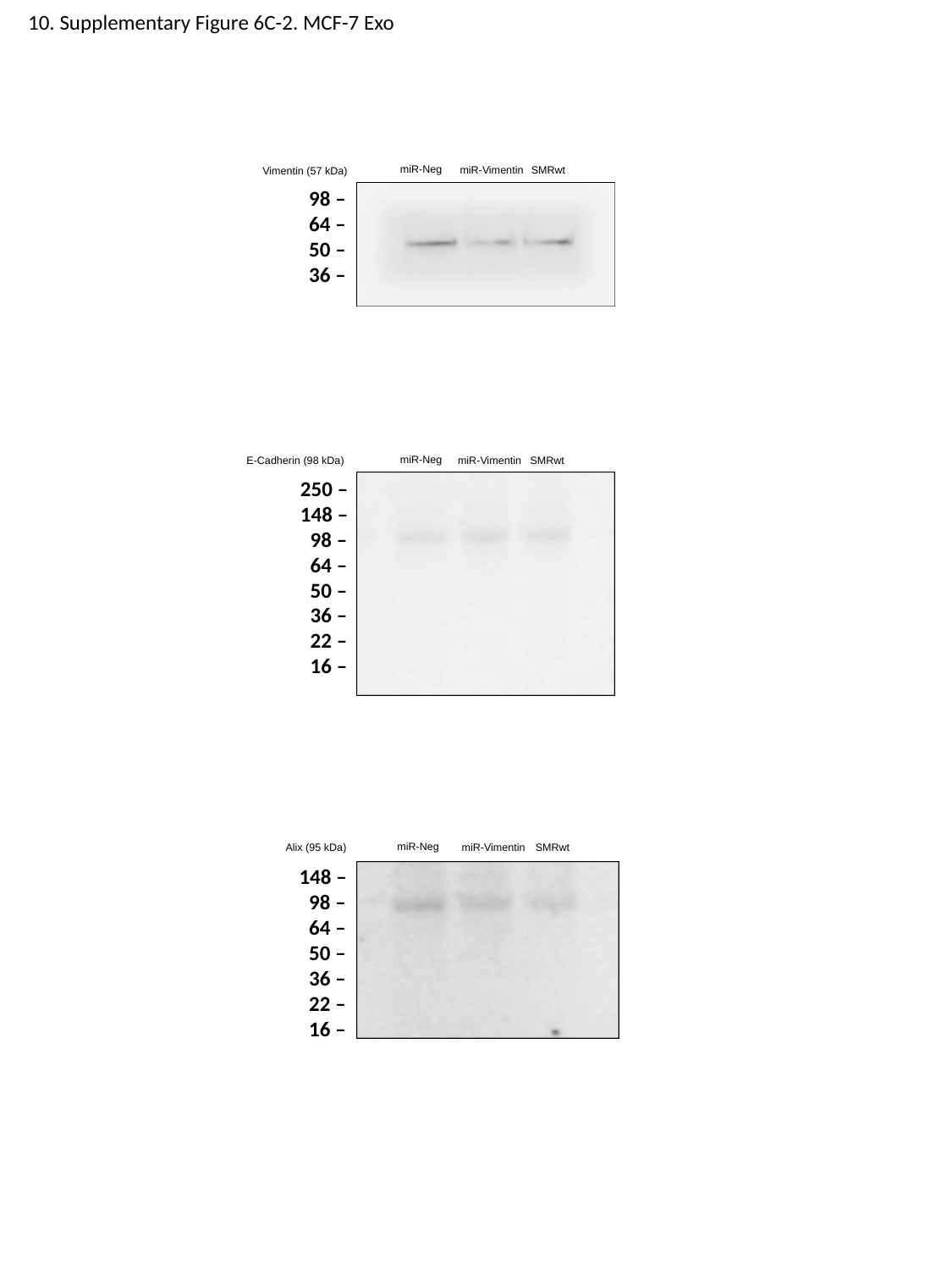

10. Supplementary Figure 6C-2. MCF-7 Exo
miR-Neg
miR-Vimentin
SMRwt
Vimentin (57 kDa)
 98 –
 64 –
 50 –
 36 –
miR-Neg
E-Cadherin (98 kDa)
miR-Vimentin
SMRwt
250 –
148 –
 98 –
 64 –
 50 –
 36 –
 22 –
 16 –
miR-Neg
Alix (95 kDa)
miR-Vimentin
SMRwt
148 –
 98 –
 64 –
 50 –
 36 –
 22 –
 16 –

## Slide 11
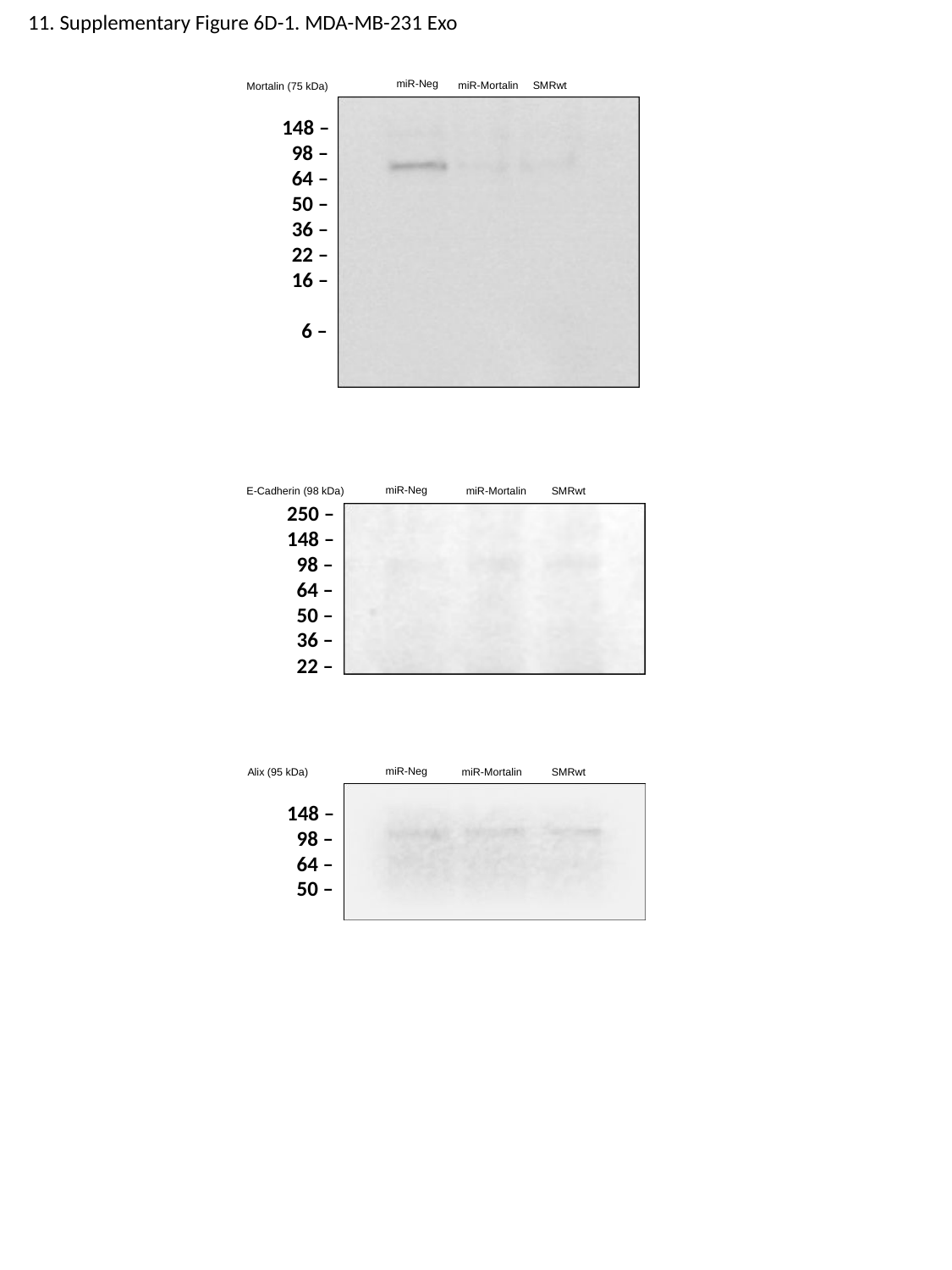

11. Supplementary Figure 6D-1. MDA-MB-231 Exo
miR-Neg
miR-Mortalin
SMRwt
Mortalin (75 kDa)
148 –
 98 –
 64 –
 50 –
 36 –
 22 –
 16 –
 6 –
miR-Neg
E-Cadherin (98 kDa)
miR-Mortalin
SMRwt
250 –
148 –
 98 –
 64 –
 50 –
 36 –
 22 –
miR-Neg
Alix (95 kDa)
miR-Mortalin
SMRwt
148 –
 98 –
 64 –
 50 –

## Slide 12
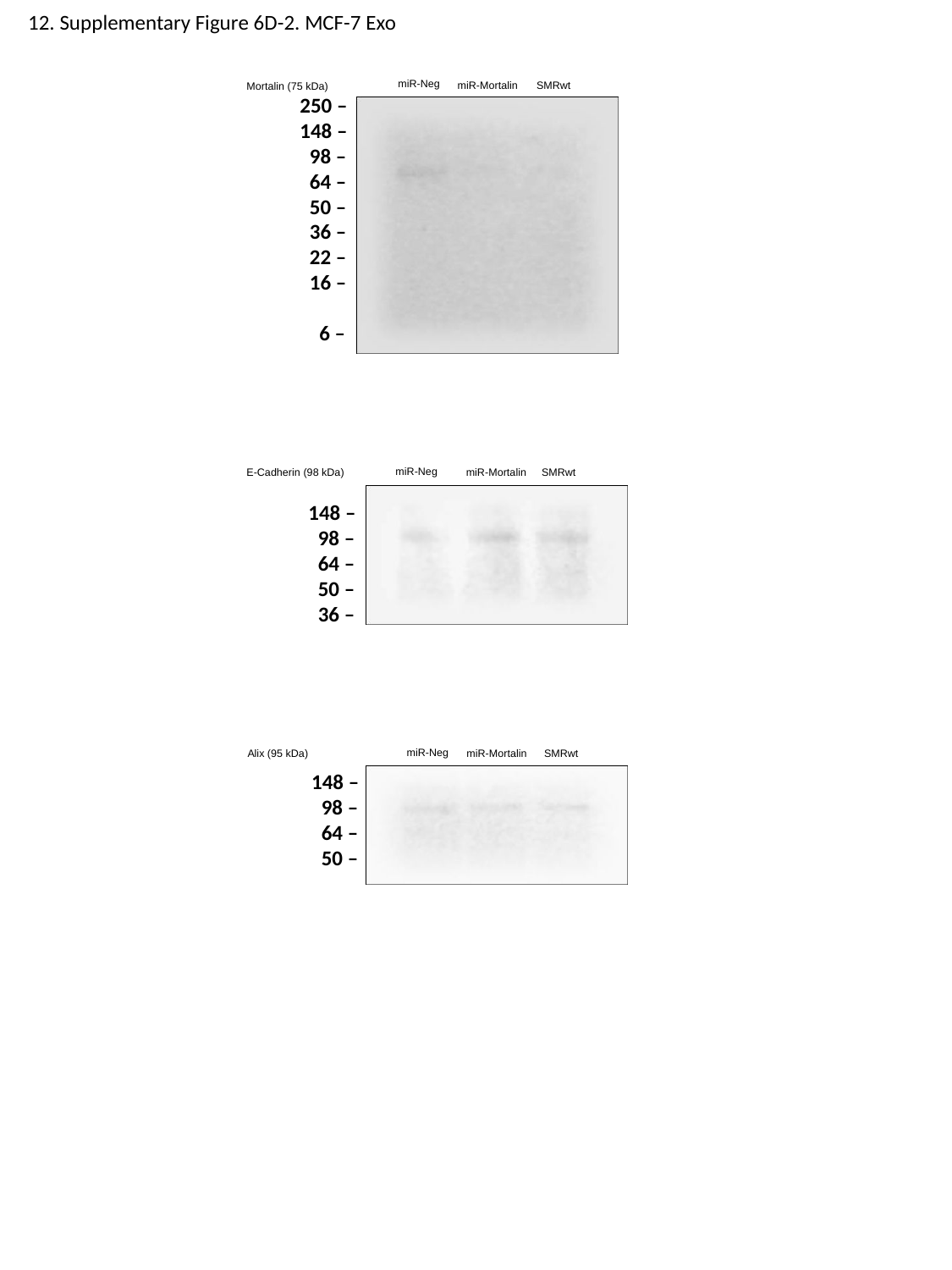

12. Supplementary Figure 6D-2. MCF-7 Exo
miR-Neg
miR-Mortalin
SMRwt
Mortalin (75 kDa)
250 –
148 –
 98 –
 64 –
 50 –
 36 –
 22 –
 16 –
 6 –
miR-Neg
E-Cadherin (98 kDa)
miR-Mortalin
SMRwt
148 –
 98 –
 64 –
 50 –
 36 –
miR-Neg
Alix (95 kDa)
miR-Mortalin
SMRwt
148 –
 98 –
 64 –
 50 –
